# Vertical Partial Laryngectomy With Temporoparietal Free‐Flap Reconstruction for Recurrent Laryngeal Cancer: Long‐Term Study

**DOI:** 10.1002/oto2.179

**Published:** 2024-08-18

**Authors:** Sharon Tzelnick, John R. de Almeida, Ralph Gilbert, David Goldstein

**Affiliations:** ^1^ Department of Otolaryngology–Head and Neck Surgery, Princess Margaret Cancer Centre University Health Network Toronto Ontario Canada; ^2^ Department of Surgical Oncology, Princess Margaret Cancer Centre University Health Network Toronto Ontario Canada

**Keywords:** outcomes, radiation, salvage, vertical partial laryngectomy

## Abstract

**Objective:**

Treatment options for recurrent early glottic carcinoma's include conservative and radical surgical options. These options offer similar survival benefits with different impacts of patient's quality of life. We previously present our experience with vertical partial laryngectomy (VPL) and showed high locoregional control rates with high‐quality voice results and normal swallowing.

**Study Design:**

A long‐term retrospective review.

**Setting:**

Tertiary Care Center.

**Methods:**

We analyzed all patients underwent VPL between the years 1995 to 2018. Long‐term oncologic and functional outcomes were collected.

**Results:**

A total of 40 patients were included. The majority of whom were male (n = 38, 95%) with a mean age of 64.9 years (SD ± 9.5). With a median follow up time of 12 years (range 0‐24), 9 patients (22.5%) had disease recurrence; the majority of whom (8 patients), had local recurrence and all were salvaged with total laryngectomy. Eight patients (20%) developed second primaries in the head and neck region with a median time to diagnosis of 77 months (range 8‐227 months). Ten‐years overall survival, disease specific survival, and local disease‐free survival were 80%, 90%, and 80%, respectively. Five patients had postoperative laryngeal dysfunction with a total 10‐years laryngectomy free survival of 70%.

**Conclusion:**

VPL has a sustainable oncologic outcome with a high long‐term laryngectomy free survival rate. This entity is an acceptable conservative salvage option for selected postradiated recurrent laryngeal squamous cell carcinoma patients.

Current management options for recurrent early glottic carcinoma (T1 and T2) includes partial laryngeal surgery (transoral endoscopic or open partial laryngectomy) or total laryngectomy (TL).^1^ While TL offers patients in the salvage surgery setting a high control rate it comes at the cost of loss of voice and requiring a permanent laryngectomy stoma, both of which can significantly alter quality of life.^2^, ^3^ While TL is a well‐recognized treatment for advanced recurrent cases, in patients with recurrent T1 or T2 tumors partial laryngeal surgical procedures, either open or endoscopic, are available to preserve the larynx. One of the issues with open salvage approaches in the postradiation setting, is the potential for poor wound healing and in order to address this our group has previously reported on a technique of salvage, reconstructed with using vascularized tissue to reconstruct salvage vertical partial laryngectomy (VPL) defects. We have demonstrated^4^ that excellent locoregional control can be achieved following VPL with a local recurrence‐free survival of 84% at 3 years and 75% at 5 years in addition to low rates of surgical complications and good functional outcomes. While early results were favorable, long‐term control and functional outcomes have not been reported. The objective of this study was to evaluate long‐term control and functional outcomes in patients with recurrent early glottic cancer that were previously treated with RT and were salvaged with VPL and temporoparietal fascia flap (TPFF).

## Methods

This study was approved by the University Health Network Research Ethics Board and the need for informed consent was waived in view of the retrospective nature of the study and no disclosure of patient identifiable information

All patients with a diagnosis of laryngeal squamous cell carcinoma (SCC) recurrence following RT that underwent salvage VPL and TPFF reconstruction, between the years 1995 to 2018, were included in this study. Our multidisciplinary approach, treatment description, and surgical technique were previously published.^4^ Following failure of RT, patients with recurrent disease staged rT1 or rT2 or small volume rT3 were considered for organ preservation approaches. Contraindication to partial surgery included laryngeal framework or cricoid involvement, disease extension outside the larynx, or pulmonary comorbidity that would preclude open surgery.

### Surgical Procedure

The TPFF is used as a vascular carrier for a buccal mucosa graft and a nonvascularized cartilage graft (usually a cartilaginous strut harvested from the contralateral thyroid ala). A TPFF measured 10 cm long × 5 cm wide is harvested with a vascular pedicle based on the superficial temporal artery and venae comitantes. The mucosal graft is thinned and sutured to the luminal surface of the TPFF. The flap is insetted and the cartilage graft is attached on the lateral aspect of the TPFF with fixation to the posterior remnant of the thyroid ala and TPFF at the level of the contralateral vocal fold. The vascular pedicle is then rotated between the ipsilateral sternohyoid and sternothyroid muscles and approximated to the carotid sheath for microvascular anastomosis which is then performed. A prefabricated Montgomery laryngeal luminal stent is inserted and held in position with transcutaneous sutures for 10 to 14 days.

### Analysis

Recurrence was documented as the date of first manifestation of recurrent disease and further subclassified as local (at the primary tumor site), regional (at the site of cervical metastases), both (locoregional), or distant (to distant organs or lymph nodes). Time to disease recurrence was documented as the number of months between the patient's surgery with VPL and TPFF reconstruction and the first noted evidence of disease recurrence. Second primary was documented as disease at another head and neck site or laryngeal cancer diagnosed 5 years after surgery.

Functional outcomes were documented as laryngeal preservation rate and swallowing status. Unserviceable larynx was defined as patients that required a tracheostomy or laryngectomy for functional purposes rather than disease recurrence. Laryngectomy free survival was defined as months from VPL. Tracheostomy rate for laryngeal stenosis was documented as well. Swallowing status was recorded as oral intake and g‐tube dependency.

Statistical analysis was performed with SPSS software, version 21.0 (IBM Corp). Categorical variables are described by frequency and percentage. Normally distributed variables are described by mean and standard deviation, and abnormally distributed variables, by median and range. Local disease‐free survival, disease specific survival and overall survival were estimated using Kaplan‐Meier method and log‐rank test.

## Results

A total of 40 patients were included in this study. Our early study group also included 40 patients; Since April 2010, we added 5 new patients to our cohort, however, long‐term analysis was available only for 40 patients as 5 patients were lost to follow up. The majority were male (n = 38, 95%) with a mean age of 64.9 years (SD ± 9.5). At their presentation with recurrence, the number of patients that staged as recurrence T1a, T1b, T2, and T3 disease, were 10 (28.5%), 7 (20%), 17 (48.5%), and 1 (3%), respectively.

### Disease Recurrence and Survival

With a median follow‐up time of 12 years (range 0‐24), 9 patients (22.5%) had disease recurrence. Of these, 8 patients (20%) had local recurrence, with a median time to recurrence of 10.5 months (range 1‐47 months) and a single patient developed lung metastasis 13 months from surgery. Of the patients that had local recurrence, 3 of the 7 that were amenable for salvage total underwent salvage surgery.

Over the follow up period, 25 patients had no evidence of disease. A total of 15 patients died; of which, 4 patients (10%) died of disease within a median of 40 months (range 14‐56) from salvage surgery. Eleven patients died of other causes with a median of 125 months (range 17‐199).

**Figure 1:**
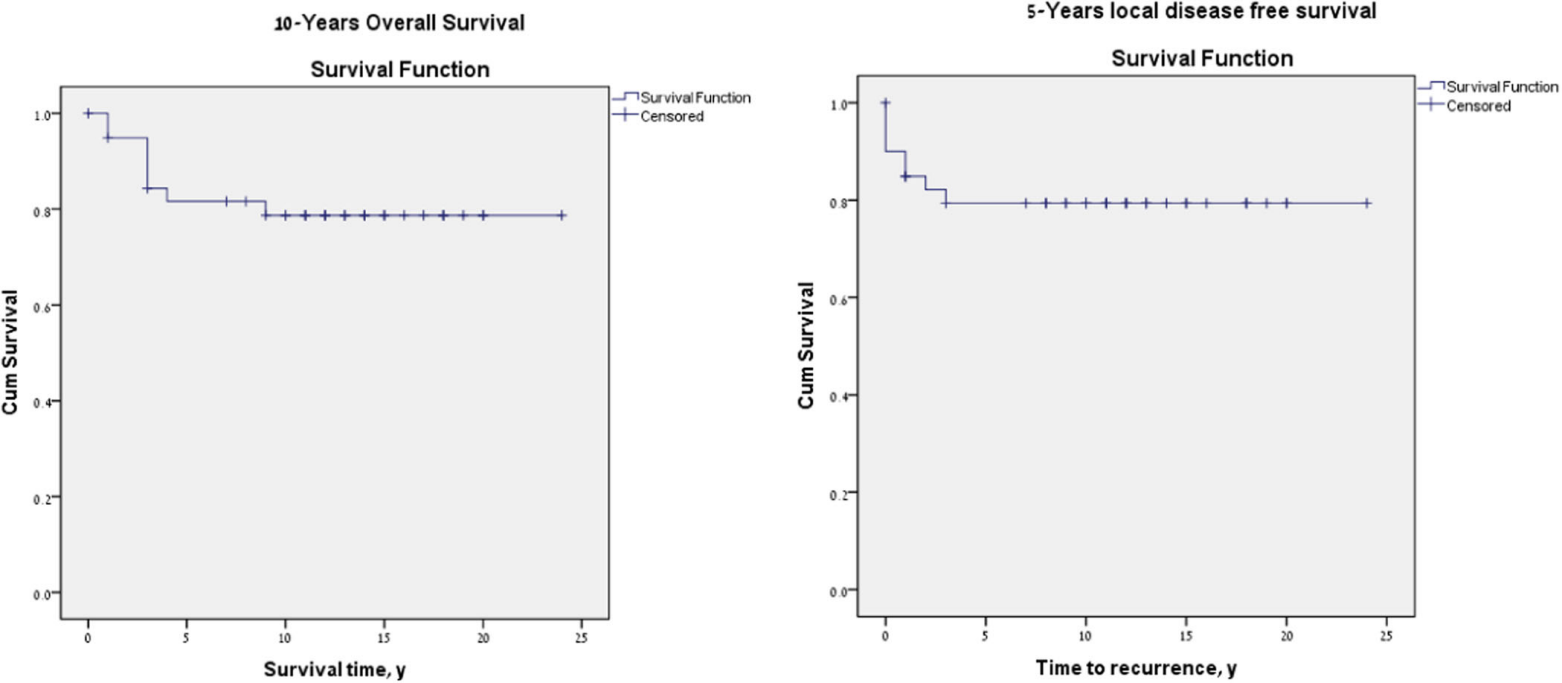


The 10‐year local control rate was 80%. Ten‐year overall survival following salvage VPL was 80%, with 10‐year disease specific survival of 90%. Ten‐year local recurrence‐free survival was 80% (Figure 1).

Eight patients (20%) developed second primaries of the head and neck region with a median time to diagnosis of 77 months (range 8‐227 months). Of these, 2 had esophageal SCC within 8 and 22 months from surgery. All other patients were diagnosed after 5 years from surgery with a diagnosis of glottis SCC (a single patient), subglottic SCC (3 patients), postcricoid SCC (a single patient), and floor of mouth SCC (a single patient). All 5 patients that had second primary in the glottis, subglottic and postcricoid area were treated with salvage TL or total laryngopharyngectomy.

### Laryngeal Disfunction and Swallowing Data

**Figure 2:**
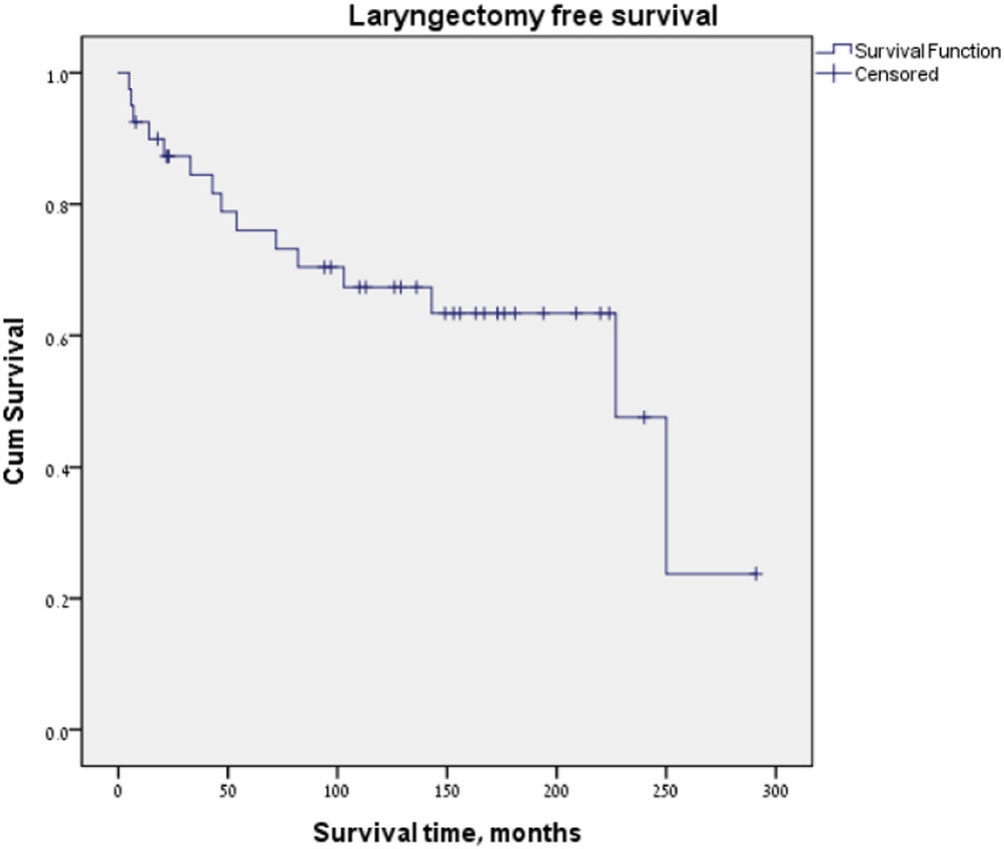


A total of 5 patients developed laryngeal stenosis and dysfunction. One patient was diagnosed with laryngeal stenosis 4 months after surgery, and was treated successfully with 3 serial dilatations. Four patients had unserviceable larynx (range of 8 months to 14.5 years postoperative), that required a tracheostomy in 1 patient and a TL in 3 patients. In total over the time period of the study, there were 12 patients that underwent a TL (3 for locoregional recurrence, 5 for second primary disease, and 3 for laryngeal dysfunction), with an overall 10‐year laryngectomy free survival of 70% (Figure 2). In terms of feeding, all patients had full oral intake. No patients were g‐tube dependent.

## Discussion

TL stands as the predominant treatment for recurrent laryngeal SCC after failing primary radiation.^5^, ^6^, ^7^ Alternative modalities like open partial laryngectomy and transoral laser surgery exist for recurrent early T1‐2 glottic SCC, providing viable options for select patients. Conservative salvage surgery, with its advantages of speech preservation and avoidance of a permanent stoma, is weighed against potential risks, particularly poor or delayed wound healing in the postradiation setting.

We have previously described and presented our approach to managing recurrent early‐stage larynx cancer after radiation with VPL and reconstruction with a temporoparietal free flap, cartilage graft, and buccal mucosal graft. We reported high locoregional control rates with a local recurrence‐free survival of 84% at 3 years in the selected patients undergoing this procedure. In this long‐term review of our oncologic and functional outcome in this patient group, we have shown a 10‐years overall survival, local disease‐free survival, and laryngectomy free survival of 80%, 80%, and 70%, respectively.

Survival data from studies on salvage partial laryngectomy substantially vary in the literature with 5‐year OS and RFS rates ranging from 52% to 95%.^8^, ^9^, ^10^, ^11^, ^12^, ^13^, ^14^, ^15^, ^16^, ^17^, ^18^, ^19^, ^20^ This include both transoral laser surgery and supracricoid partial laryngectomy approaches. Our study results show an 80% 5‐year overall survival and recurrence‐free survival rate in patients that have early primary glottic disease and early stages at recurrence as well. Laryngeal recurrence diagnosed beyond 5 years from treatment was considered as second primary, and a total of 5 patients (12.5%) had laryngeal second primaries that included the glottis; subglottic and postcricoid area. Notably, all patients diagnosed with laryngeal second primaries underwent salvage laryngectomy. This finding underscores the elevated risk of additional head and neck cancers in this cohort, aligning with existing literature indicating a second primary incidence of 2% to 6% per year and a cumulative rate of 36% at 20 years among patients with head and neck SCC.^21^ Examining those with disease recurrence or laryngeal second primaries, 61.5% underwent salvage laryngectomy, resulting in no further disease recurrence during long‐term follow‐up.

A recent meta‐analysis by Shapira et al^22^ examining salvage partial laryngectomy after radiation failure presented an overall laryngectomy free survival rate of 81.2%, with 90.4% for open partial laryngectomy and 78.6% for the transoral laser surgery. However, these rates are a representative of a minimum follow up time between 2 and 5 years, with no follow‐up range of years or maximum follow‐up time of all included studies. Our results showed a long‐term 70% laryngectomy free survival with a median follow up of 12 years. Of the patients that underwent salvage TL, 4 patients had unserviceable larynx that needed TL or tracheostomy, however a number of these were able to avoid a stoma for a long time with the range of 8 months to 14 years. Although constituting a small percentage (7.5%), this highlights the importance of prolonged functional follow‐up and screening for laryngeal second primaries in this patient subset.

In conclusion, our findings emphasize the sustainable oncologic outcomes of VPL, offering a high long‐term laryngectomy‐free survival rate. This conservative salvage option emerges as an acceptable and effective choice for carefully selected patients with recurrent laryngeal SCC postradiation, showcasing promising prospects for both survival and functional outcomes. While recurrence is infrequent these patients do need to be followed for the development of a second primary as well as the subset of patients that can develop laryngeal stenosis.

## Author Contributions

**Sharon Tzelnick**, acquisition and analysis of data, drafting the article and final approval of the version to be published; **John R. de Almeida**, revising the manuscript and final approval of the version to be published; **Ralph Gilbert**, substantial contributions to conception and design, revising the manuscript and final approval of the version to be published; **David Goldstein**, substantial contributions to conception and design, revising the manuscript and final approval of the version to be published.

## Disclosures

### Competing interests

None declared.

### Funding source

This research did not receive any specific grant from funding agencies in the public, commercial, or not‐for‐profit sectors.

## References

[1] Vander Poorten V , Meulemans J , Beitler JJ , et al. Salvage surgery for residual or recurrent laryngeal squamous cell carcinoma after (chemo)radiotherapy: oncological outcomes and prognostic factors. Eur J Surg Oncol. 2021;47(11):2711‐2721. 10.1016/j.ejso.2021.05.035 34059377

[2] Zenga J , Goldsmith T , Bunting G , Deschler DG . State of the art: rehabilitation of speech and swallowing after total laryngectomy. Oral Oncol. 2018;86:38‐47. 10.1016/j.oraloncology.2018.08.023 30409318

[3] Wulff NB , Dalton SO , Wessel I , et al. Health‐related quality of life, dysphagia, voice problems, depression, and anxiety after total laryngectomy. Laryngoscope. 2022;132(5):980‐988. 10.1002/lary.29857 34490903

[4] Gilbert RW , Goldstein DP , Guillemaud JP , Patel RS , Higgins KM , Enepekides DJ . Vertical partial laryngectomy with temporoparietal free flap reconstruction for recurrent laryngeal squamous cell carcinoma. Archi Otolaryngol Head Neck Surg. 2012;138(5):484. 10.1001/archoto.2012.410 22652947

[5] Haapaniemi A , Väisänen J , Atula T , Alho OP , Mäkitie A , Koivunen P . Predictive factors and treatment outcome of laryngeal carcinoma recurrence. Head Neck. 2017;39(3):555‐563. 10.1002/hed.24642 27902867

[6] Ganly I , Patel SG , Matsuo J , et al. Results of surgical salvage after failure of definitive radiation therapy for early‐stage squamous cell carcinoma of the glottic larynx. Arch Otolaryngol Head Neck Surg. 2006;132(1):59‐66. 10.1001/archotol.132.1.59 16415431

[7] Mimica X , Hanson M , Patel SG , et al. Salvage surgery for recurrent larynx cancer. Head Neck. 2019;41(11):3906‐3915. 10.1002/hed.25925 31433540 PMC7485603

[8] Gigot M , Digonnet A , Rodriguez A , Lechien JR . Salvage partial laryngectomy after failed radiotherapy: oncological and functional outcomes. J Clin Med. 2022;11(18):5411. 10.3390/jcm11185411 36143058 PMC9500615

[9] Schwaab G , Mamelle G , Lartigau E , Parise O , Wibault P , Luboinski B . Surgical salvage treatment of T1/T2 glottic carcinoma after failure of radiotherapy. Am J Surg. 1994;168(5):474‐475. 10.1016/S0002-9610(05)80104-7 7977978

[10] Yiotakis J , Stavroulaki P , Nikolopoulos T , et al. Partial laryngectomy after irradiation failure. Otolaryngol Head Neck Surg. 2003;128(2):200‐209. 10.1067/mhn.2003.63 12601315

[11] Makeieff M , Venegoni D , Mercante G , Crampette L , Guerrier B . Supracricoid partial laryngectomies after failure of radiation therapy. Laryngoscope. 2005;115(2):353‐357. 10.1097/01.mlg.0000154751.86431.41 15689765

[12] Pellini R , Pichi B , Ruscito P , et al. Supracricoid partial laryngectomies after radiation failure: a multi‐institutional series. Head Neck. 2008;30(3):372‐379. 10.1002/hed.20709 17972314

[13] Toma M , Nibu K , Nakao K , et al. Partial laryngectomy to treat early glottic cancer after failure of radiation therapy. Arch Otolaryngol Head Neck Surg. 2002;128(8):909. 10.1001/archotol.128.8.909 12162769

[14] Philippe Y , Espitalier F , Durand N , Ferron C , Bardet E , Malard O . Partial laryngectomy as salvage surgery after radiotherapy: oncological and functional outcomes and impact on quality of life. A retrospective study of 20 cases. Eur Ann Otorhinolaryngol Head Neck Dis. 2014;131(1):15‐19. 10.1016/j.anorl.2012.11.008 24139073

[15] Rodríguez‐Cuevas S , Labastida S , Gonzalez D , Briseño N , Cortes H . Partial laryngectomy as salvage surgery for radiation failures in T1–T2 laryngeal cancer. Head Neck. 1998;20(7):630‐633. 10.1002/(SICI)1097-0347(199810)20:7<630::AID-HED9>3.0.CO;2-K 9744464

[16] Quer M , León X , Orús C , Venegas P , López M , Burgués J. Endoscopic laser surgery in the treatment of radiation failure of early laryngeal carcinoma. Head Neck. 2000;22(5):520‐523. 10.1002/1097-0347(200008)22:5<520::AID-HED13>3.0.CO;2-K 10897114

[17] Barbu AM , Burns JA , Lopez‐Guerra G , Landau‐Zemer T , Friedman AD , Zeitels SM . Salvage endoscopic angiolytic KTP laser treatment of early glottic cancer after failed radiotherapy. Ann Otol Rhinol Laryngol. 2013;122(4):235‐239. 10.1177/000348941312200404 23697320

[18] Weiss BG , Bertlich M , Canis M , Ihler F . Transoral laser microsurgery or total laryngectomy for recurrent squamous cell carcinoma of the larynx: retrospective analysis of 199 cases. Head Neck. 2017;39(6):1166‐1176. 10.1002/hed.24737 28252821

[19] Del Bon F , Piazza C , Mangili S , Redaelli De Zinis LO , Nicolai P , Peretti G . Transoral laser surgery for recurrent glottic cancer after radiotherapy: oncologic and functional outcomes. Acta Otorhinolaryngol Ital. 2012;32(4):229‐237. 23093812 PMC3468937

[20] De Virgilio A , Pellini R , Mercante G , et al. Supracricoid partial laryngectomy for radiorecurrent laryngeal cancer: a systematic review of the literature and meta‐analysis. Eur Arch Otrhinolaryngol. 2018;275(7):1671‐1680. 10.1007/s00405-018-4986-4 29713885

[21] Morris LG , Sikora AG , Hayes RB , Patel SG , Ganly I . Anatomic sites at elevated risk of second primary cancer after an index head and neck cancer. Cancer Causes Control. 2011;22(5):671‐679. 10.1007/s10552-011-9739-2 21327458 PMC3085084

[22] Shapira U , Warshavsky A , Muhanna N , et al. Laryngectomy‐free survival after salvage partial laryngectomy: a systematic review and meta‐analysis. Eur Arch Otrhinolaryngol. 2022;279(6):3021‐3027. 10.1007/s00405-022-07257-2 35039895

